# Integrity and Stability of PTC Bearing CFTR mRNA and Relevance to Future Modulator Therapies in Cystic Fibrosis

**DOI:** 10.3390/genes12111810

**Published:** 2021-11-18

**Authors:** Luka A. Clarke, Vanessa C. C. Luz, Szymon Targowski, Sofia S. Ramalho, Carlos M. Farinha, Margarida D. Amaral

**Affiliations:** BioISI—Biosystems & Integrative Sciences Institute, Faculty of Sciences, University of Lisbon, 1749-016 Lisbon, Portugal; vanessac.luz.25@gmail.com (V.C.C.L.); szymon.targowski@gumed.edu.pl (S.T.); ssramalho@fc.ul.pt (S.S.R.); cmfarinha@fc.ul.pt (C.M.F.); msamaral@fc.ul.pt (M.D.A.)

**Keywords:** cystic fibrosis, CFTR, PTC mutations, nonsense-mediated decay, readthrough

## Abstract

Major advances have recently been made in the development and application of CFTR (cystic fibrosis transmembrane conductance regulator) mutation class-specific modulator therapies, but to date, there are no approved modulators for Class I mutations, i.e., those introducing a premature termination codon (PTC) into the CFTR mRNA. Such mutations induce nonsense-mediated decay (NMD), a cellular quality control mechanism that reduces the quantity of PTC bearing mRNAs, presumably to avoid translation of potentially deleterious truncated CFTR proteins. The NMD-mediated reduction of PTC-CFTR mRNA molecules reduces the efficacy of one of the most promising approaches to treatment of such mutations, namely, PTC readthrough therapy, using molecules that induce the incorporation of near-cognate amino acids at the PTC codon, thereby enabling translation of a full-length protein. In this study, we measure the effect of three different PTC mutations on the abundance, integrity, and stability of respective CFTR mRNAs, using CFTR specific RT-qPCR-based assays. Altogether, our data suggest that optimized rescue of PTC mutations has to take into account (1) the different steady-state levels of the CFTR mRNA associated with each specific PTC mutation; (2) differences in abundance between the 3′ and 5′ regions of CFTR mRNA, even following PTC readthrough or NMD inhibition; and (3) variable effects on CFTR mRNA stability for each specific PTC mutation.

## 1. Introduction

Cystic Fibrosis (CF) is an autosomal recessive condition caused by mutations in the CF transmembrane conductance regulator (*CFTR*) gene [1], which encodes a chloride/bicarbonate channel important for maintaining ion homeostasis across epithelial membranes, perhaps most essentially in the airways [2]. Of the more than 2100 potentially disease-causing mutations in *CFTR*, approximately 8.4% are nonsense mutations, i.e., single base pair substitutions, which introduce premature termination codons (PTCs) into the CFTR coding sequence [3]. Nonsense mutations, which are classified as Class I mutations in CF, are associated with severe disease phenotypes [4] and have two major effects upon CFTR expression. Firstly, the presence of a PTC causes the translation of a truncated CFTR protein, and such proteins are generally nonfunctional, if not deleterious, with few exceptions. Indeed, the only ones retaining some chloride channel function potentially rescuable by CFTR modulators, are those in which the PTC occurs from around exon 23 onwards, as in the case of the W1282X mutation [5,6]. Secondly, most PTCs trigger nonsense-mediated decay (NMD), a cellular quality control mechanism that specifically accelerates the degradation of mRNAs bearing PTCs [7], thereby reducing the production of truncated and nonfunctional (potentially deleterious) protein. Nonsense mutations in *CFTR* are therefore associated with a marked reduction in CFTR mRNA [8], and an almost complete elimination of functional protein production [9].

The effects of NMD have, however, been shown to vary among different cell types [10] and we have shown that while NMD is responsible for degradation of up to 80% of PTC-bearing CFTR transcripts, there is variation not only between different tissues (nasal epithelial cells and intestinal organoids), but also among both different mutations and individuals with the same genotype [11]. Such variations in NMD have been shown to affect individual responses to one of the most promising therapeutic approaches for nonsense mutations, namely, PTC readthrough [12]. The quantity of remaining PTC-bearing mRNAs in such studies, which is generally 20–30% of measurable total CFTR transcripts, is well above the threshold at which functional CFTR protein can be produced [13]. It is, however, not known to what extent this residual mRNA exists in the form of full-length transcripts, suitable for readthrough and translation of full-length protein, or is rather a pool of semidigested fragments [14], since PTC-bearing mRNA in a living cell is the product of a dynamic balance of continuous transcription, coupled with degradation via the NMD pathway. NMD itself is triggered during the first round of translation in the ribosome, when an exon junction complex (EJC) is detected downstream of the PTC [15]. The PTC-bearing transcript is then subject either to SMG6-mediated cleavage near the site of the PTC itself, followed by exonuclease digestion from both ends of the resulting fragments [16,17], or to SMG5/7-mediated exonucleolytic decay following deadenylation and decapping of the mRNA [18]. It is therefore likely that many PTC-bearing mRNA transcripts detected are partial, being an incomplete result of these processes, and may therefore not be suitable for the translation of full-length proteins in the context of PTC readthrough.

PTC mutations represent one of the last major groups of CF causing defects for which a specific modulator therapy has not yet been approved [19]. An effective therapeutic approach to the treatment of PTC mutations might take the form of a combination [20], in which an NMD inhibitor is used to enhance cellular levels of mRNA, and an effective and nontoxic readthrough molecule, then stimulates the translation of a full-length CFTR protein, albeit one that contains a near-cognate amino acid at the site of the PTC [21]. CFTR correctors and/or potentiators could then be used to stimulate both traffic and function of the rescued PTC-CFTR protein [22]. Not all PTC mutations, however, respond in the same way to such approaches, and they can be separated into subclasses based on their amenability to such rescue strategies [23]. A part of these differences might be explainable by variations in the integrity and stability of CFTR mRNAs specific to particular mutations, resulting in variable quantities of available full-length CFTR mRNAs as a template for further therapeutic rescue. 

Here, we report on the use of RT-qPCR-based assays to assess measures of integrity and stability of CFTR mRNAs bearing three different PTC mutations, namely, Y122X, G542X, and W1282X. Integrity was measured by comparing the abundance of transcripts from the 3′ and 5′ ends of the CFTR transcript, and mRNA stability was measured using transcriptional shutdown by treatment with Actinomycin-D, followed by production of decay curves and estimation of the mRNA half-life. We thereby demonstrate differences in the integrity and stability of transcripts bearing those three mutations in *CFTR*, and the effects of NMD inhibition and PTC readthrough upon these measures. These differences may provide clues to understanding mutation-specific responses to PTC correction therapies.

## 2. Materials and Methods

### 2.1. Cell Lines and Treatments

16HBE14o-bronchial epithelial cell lines (henceforth termed 16HBE cells) stably expressing a single copy of *CFTR* genes bearing the mutations c.366T>A, c.1624G>T, and c.3846G>A (legacy names: Y122X, G542X, and W1282X, respectively) were generously provided by Dr. M. Mense from the CFFT Laboratory [24]. 16HBE cells expressing wild-type (wt) *CFTR* were used as a control. For the sake of clarity and familiarity, the legacy names of the three PTC mutations have been used throughout this article. The SMG1i inhibitor compound (M.W.: 566.07 g/mol) was also kindly provided by Dr. Mense and administered for 24 h at a final concentration of 0.5 or 1 μM from an initial stock of 10 mM in DMSO. G418 sulfate (Gibco, Thermo Fisher Scientific, Paisley, UK) was dissolved in water (50 mg/mL stock) and added to cells for 24 h at a final concentration of 250 or 500 μg/mL. Although the aminoglycoside G418 is primarily known in this context as an experimental PTC readthrough agent, it has also been observed to enhance the expression of PTC-bearing mRNAs [25], similarly to an NMD inhibitor. Since mRNA-associated EJC proteins are required for the triggering of NMD [15], translational readthrough by G418 is expected to remove this trigger, thereby reducing NMD-associated depletion of the PTC-bearing mRNA. The enhancement of mRNA abundance by G418 is thus likely to be due to evasion of NMD, and this effect has been found to be synergistic to the direct NMD inhibitory effect of SMG1i [20], supporting the existence of distinct mechanisms of action.

### 2.2. RNA Isolation, Reverse Transcription of cDNA

Total RNA was isolated from cell samples by addition of TRIzol reagent (Invitrogen, Thermo Fisher Scientific, Merelbeke, Belgium) and using the Zymo Research kit (Direct-zol™ RNA Miniprep Plus, Zymo Research, Irvine, CA, USA). Extraction by this method incorporates a DNase I digestion step to eliminate traces of genomic DNA from the RNA sample. Complementary DNA (cDNA) was reverse transcribed (RT) from a maximum of 1 µg total RNA in a volume of 20 µL using mmlv RT (NZYtech Ltd., Lisboa, Portugal) and random primers. For PCR amplification, cDNA samples were diluted 1:10 in nuclease-free water.

### 2.3. Primers

**Figure 1:**
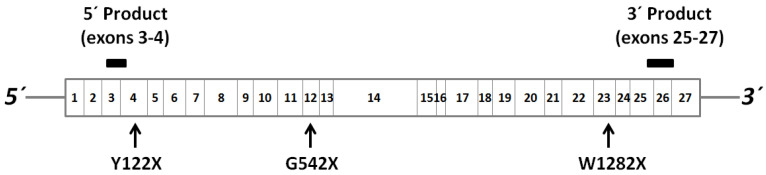
Positions of 5′ and 3′ PCR products amplified from CFTR cDNA in the present study (black bars). The positions of the three PTC mutations studied are also shown. The currently used CFTR exon numbers are given, according to the CFTR mutation database [3].

Primer pairs from the 5′ and 3′ ends of the CFTR cDNA sequence were used for amplification of PCR products in exons 3–4 (CFTR-5′, 127 bp: forward primer 5′-TGCCCTTCGGCGATGTTTTT-3′; reverse primer 5′-GTTATCCGGGTCATAGGAAGCTA-3′) and exons 25–27 (CFTR-3′, 180 bp: forward primer 5′-GCGAAGATCTTGCTGCTTGA-3′; reverse primer 5′-CTGCCGCACTTTGTTCTCTT-3′). These primer sequences were originally obtained from the Harvard University Primerbank [26] and were paired due to their having similar amplification efficiencies (and the closest to 100%) calculated using the CFX Maestro software (Bio-Rad, Hercules, CA, USA) following the production of RT-qPCR standard curves for each primer pair. The positions of the primer pairs relative to the PCR mutations studied are shown in Figure 1. For RNA shutdown assays, the CFTR-3′ product was initially used. β actin (ACTB) was used as a housekeeping gene for normalization of RT-qPCR data. 

### 2.4. Real-Time Quantitative RT-PCR

To determine relative quantitative abundance of CFTR mRNA transcripts, we used the ddCT RT-qPCR method. Products were amplified with technical duplicates or triplicates, for at least *n* = 3 independent experiments, using the SsoFast EvaGreen system (Bio-Rad, Hercules, CA, USA). CT values for CFTR were normalized using CT values for the reference gene ACTB amplified in the same samples, and mean fold change (FC) between experimental and control conditions was calculated using the formula FC = 2^(−ddCT)^, as previously described [11].

### 2.5. mRNA Integrity Assay

The mRNA integrity assay used here was developed to determine the relative abundance of fragments of CFTR transcript at both 3′ and 5′ ends, and the ratio between them. It is a simple measure of the likely integrity of the CFTR mRNA, and the relative degradation occurring at either end. Previous 3′:5′ integrity assays have used oligo dT priming of cDNA in order to determine overall mRNA integrity measured from the 3′ end [27,28], but we used random primers to more accurately reflect the proportion of transcript fragments that include sequence from the 5′ end, and not only those that include the poly-A tail. This allowed us to produce a rough estimate of the proportion of total transcripts from which protein translation might be initiated, therefore, providing an integrity measurement with some functional significance. For example, a 3′:5′ ratio of 3 would imply that a 1:3 proportion of total CFTR encoding transcripts (i.e., 25%) contained a sequence from the 5′ end. The ratio between 3′ and 5′CFTR mRNA abundance was calculated using RT-qPCR amplification of both CFTR products in *n* = 3 samples (except for the wt-CFTR control conditions, in which *n* = 2 samples were used), using the formula FC = 2^^(−ddCT)^, as described above.

### 2.6. mRNA Stability Assay

Stability of *CFTR* mRNA was estimated by administration of Actinomycin-D (5 µg/mL, Gibco, Thermo Fisher Scientific, Waltham, MA, USA), which has long been known to be an inhibitor of RNA-Pol-II-mediated RNA synthesis [29], and subsequent RT-qPCR-based measurements of CFTR mRNA abundance relative to the time of administration over the following 12 h. ACTB was used as the best control gene for normalization, following comparative optimization of the assay using other controls (18S-rRNA, RPLP0, and CFTR itself at t = 0 h), and considering the higher than average stability of ACTB mRNA [30]. For the purposes of curve fitting, we assumed exponential mRNA degradation following transcriptional shutdown according to the formula y = (1/B)·e^((A−x)/B)^, where A and B are position and scale parameters, respectively. Degradation curves were constructed by best fit using the least squares method in the excel add-on solver [31], and approximate half-lives of CFTR mRNA were estimated.

### 2.7. Western Blot (WB) Analysis

For CFTR protein detection, 16HBE cells were lysed in Laemmli buffer supplemented with complete protease inhibitor (PIC) tablets (Roche, Basel, Switzerland). Lysates were separated by SDS-PAGE and transferred to a PVDF membrane (Millipore, Burlington, MA, USA). CFTR was detected using the anti-CFTR monoclonal antibody (mAb) TJA9 (CFF) recognizing amino acids 103–117 (i.e., first extracellular loop, EL1) [32], used at dilution 1:500 and then by reblotting with anti-CFTR 596 (CFF) recognizing amino acids 1204–1211 (NBD2) [33] at 1:3000 dilution. Secondary antibody was horseradish peroxidase-labelled antimouse at 1:3000 (BioRad). Anti-α-tubulin antibody (1:10,000) (Sigma, Darmstadt, Germany) was used as loading control. Images were acquired using ChemiDoc XRS+ imaging system BioRad and further processed by Image lab 4.0 software. All comparisons presented here are based on relative quantifications made within the same blot with equivalent amount of protein, same exposure, and comparing with a loading control.

### 2.8. Statistical Analysis

Bar charts were plotted as the mean with error bars representing the standard error of the mean (SEM). Differences between groups were assessed using two-tailed unpaired student *t* test. *p*-values conferring significance are shown in figures by varying the number of asterisks depending on the value (* *p* < 0.05; ** *p* < 0.01; *** *p* < 0.001), with any *p*-value less than 0.05 considered as statistically significant.

## 3. Results

### 3.1. PTC Mutations Cause a Reduction in CFTR mRNA Abundance

**Figure 2:**
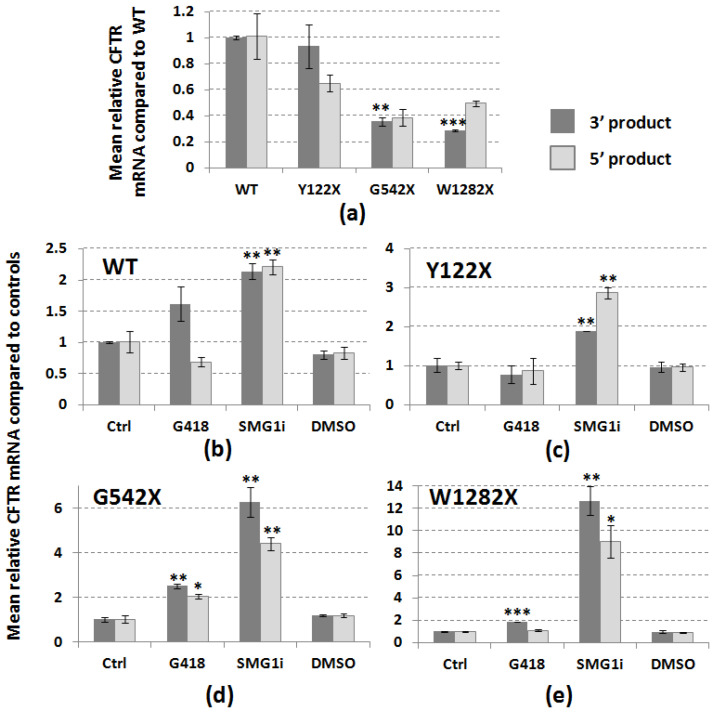
Effect of PTC mutations on abundance of CFTR mRNA and rescue using PTC readthrough and NMD inhibition. Graphs show relative quantification of CFTR mRNA amplified by RT-qPCR using primers designed at 3′ and 5′ ends (dark vs. light bars, respectively). (**a**) Relative abundance of 5′ and 3′ fragments with three different PTC mutations (Y122X, G542X, and W1282X) compared with wt-CFTR. (**b**–**e**) Effect of PTC readthrough (G418, 250 µg/mL, 24 h), NMD inhibition (SMG1i, 0.5 µM, 24 h), or DMSO (0.5%, 24 h) on abundance of 3′ and 5′ CFTR for four different genotypes (b: wt; c: Y122X; d: G542X; e: W1282X). Ctrl = untreated cells. Values shown are means (*n* = 3, except for wt-Ctrl: *n* = 2) ±SEM, and significant differences from respective control conditions are indicated by asterisks (* *p* < 0.05; ** *p* < 0.01; *** *p* < 0.001).

Data on relative abundance of CFTR mRNA were generated by RT-qPCR using primers designed towards the 3′ and 5′ ends of the CFTR mRNA transcript in 16HBE cells expressing either wt-CFTR or CFTR with one of three different PTC mutations (Figure 1). As expected, based on our previously published data [11], the presence of any one of the PTC mutations—Y122X, G542X, or W1282X—was associated with a reduction in abundance of CFTR mRNA, which was significant for each of the products amplified at the 3′ end (Figure 2a; *p* < 0.01; *n* = 3). For these 3′ RT-PCR amplicons, which represented a fragment encompassing parts of exons 25–27, there was a progressive decrease in relative CFTR mRNA abundance the further towards the 3′ end the PTC mutation was situated.

### 3.2. PTC Readthrough and NMD Inhibition Can Restore PTC-Associated CFTR mRNA Levels

In Figure 2b–e, we present data on the relative abundance of CFTR mRNA measured by RT-qPCR amplifying 3′ and 5′ CFTR products from 16HBE cells treated for 24 h with either the PTC readthrough inducing aminoglycoside compound G418/Geneticin (250 μg/mL), the SMG1i inhibitor (0.5 μM), or DMSO (0.5%) as a control for the SMG1i vehicle. No significant differences in CFTR expression were observed following DMSO treatment in cells with any of the four CFTR genotypes (Figure 2b–e). However, treatment with SMG1i induced significant increases (*p* < 0.05 or *p* < 0.01) in CFTR mRNA for both 3′ and 5′ products in cells expressing wt-CFTR and each of the three PTC mutations, with progressively greater-fold-increases in CFTR expression with increasing proximity of the PTC to the 3′ end (Figure 2c–e). PTC readthrough induced by G418 treatment also led to increases in CFTR mRNA abundance, although to a much lesser extent than for SMG1i, and the differences were only found to be significant for the 3′ and 5′ products of G542X (*p* < 0.01 and *p* < 0.05, respectively), and the 3′ product of W1282X (*p* < 0.001). Reanalysis of these data using values from untreated cells expressing wt-CFTR as a baseline shows that treatment with SMG1i significantly raised CFTR mRNA abundance above the baseline for all genotypes at the 3′ end (Figure S1). A similar pattern was seen for the 5′ product, but this only reached significance for the W1282X mutation, and neither G418 nor DMSO treatments raised the CFTR mRNA abundance above control levels.

### 3.3. PTC Readthrough and NMD Inhibition Do Not Significantly Restore CFTR Protein Expression

**Figure 3:**
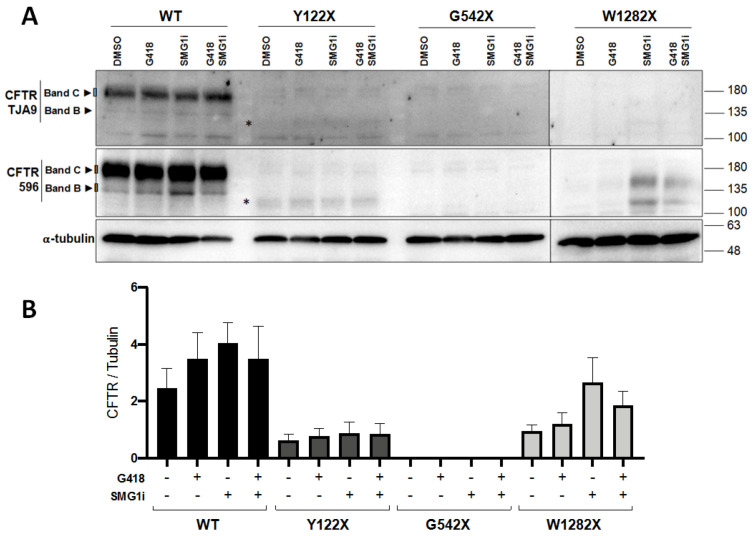
Expression of CFTR protein following treatment with G418 and/or SMG1i. (**A**) Representative Western Blot analysis of CFTR protein expression in 16HBE cells expressing wt-CFTR or Y122X, G542X, and W1282X-CFTR mutations following treatment with G418 (500 µg/mL) and SMG1i (1 µM) for 24 h. CFTR was detected using first the anti-CFTR TJA9 (1:500), which recognizes the ECL1 domain (upper panel), and then reblotted with the anti-CFTR 596 (1:3000), which recognizes the NBD2 domain (middle panel). Images were acquired using ChemiDoc XRS+ imaging system (BioRad) and further processed by Image lab 4.0 software (*n* = 3). (**B**) For blots incubated with 596 antibody, densitometry was used to quantify total CFTR (band C + band B) and normalized to the loading control (α-tubulin). Data shown as mean ± SEM (*n* = 3). Asterisks in (**A**) indicate truncated CFTR potentially produced due to reinitiation of translation (~128 kDa).

Western blots (WB) using the anti-CFTR antibodies TJA9 and 596 (see Methods) clearly demonstrated the abolition of mature CFTR protein expression by all three PTC mutations (Figure 3A). Expression of full-length CFTR protein was not significantly restored by either separate or combined treatments with the readthrough agent G418 or the NMD inhibitor SMG1i (Figure 3B). However, in contrast to the definitive absence of any expression of G542X-CFTR, the Y122X-CFTR protein did display a residual signal (see asterisks, Figure 3A) corresponding to truncated CFTR potentially produced due to re-initiation of translation [34], and there was evidence of an increase in expression of W1282X CFTR protein following SMG1i treatment with or without G418.

### 3.4. Ratio between Abundance of CFTR Transcript at 3′ and 5′ Ends

**Figure 4:**
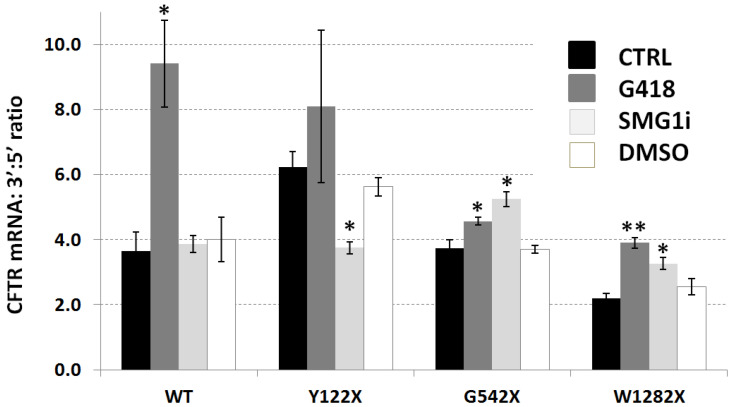
Ratios of abundance of 3′ and 5′ CFTR mRNA, measured by RT-qPCR. Ratios of 3′ to 5′ transcript abundance were determined for 16HBE cells expressing wt-CFTR and three different PTC mutations (Y122X, G542X, W1282X: x-axis) under four conditions, shown in four shades (CTRL: untreated cells; G418: 500 µg/mL, 24 h; SMG1i: 1 µM, 24 h; DMSO: 0.5%, 24 h). Values shown are means (*n* = 3) ± SEM, and significant differences from untreated cells are indicated by asterisks (* *p* < 0.05; ** *p* < 0.01).

Ratios of abundance of CFTR transcripts amplified towards the 3′ and 5′ ends in 16HBE cells were generated using RT-qPCR with two primer pairs with very similar amplification efficiencies (see Methods). The ratios obtained are presented in Figure 4. For all *CFTR* genotypes (including wt) and under all conditions, the 3′:5′ ratio was greater than 1, meaning that sections of CFTR transcript including the amplified 3′ fragment were more abundant than those containing the amplified 5′ fragment. For the three PTC mutations, there was a nonsignificant tendency for the ratio to be lower when the mutation was closer to the 3′ end. For the G542X and W1282X mutations, both G418 and SMG1i treatments significantly increased the observed 3′:5′ ratio (*p* < 0.05 or *p* < 0.01). For the Y122X mutation, SMG1i treatment led to a significant decrease in the ratio (*p* < 0.05), and the increase observed upon G418 treatment was not found to be significant. However, G418 also significantly increased the 3′:5′ ratio of wt-CFTR mRNA transcripts (*p* < 0.05), perhaps revealing a nonspecific effect of G418 in the absence of its PTC readthrough function that may also be important in Y122X where the PTC may evade the effects of NMD via reinitiation of translation [34] (see Section 4).

In order to confirm the decreased abundance of 5′ CFTR mRNA transcript in other tissues, we generated 3′:5′ ratios in native nasal epithelial cells and lung samples from healthy donors and individuals with CF (see Supplementary Data, Figure S2). In each of the four groups, the ratios, although not as high as in the 16HBE cell lines, were found to be greater than 1, thereby confirming the relevance of our data across different cell types and in the context of PTC readthrough therapy.

### 3.5. The Effect of PTC Readthrough and NMD Inhibition on Stability of PTC-Bearing CFTR mRNA

**Figure 5:**
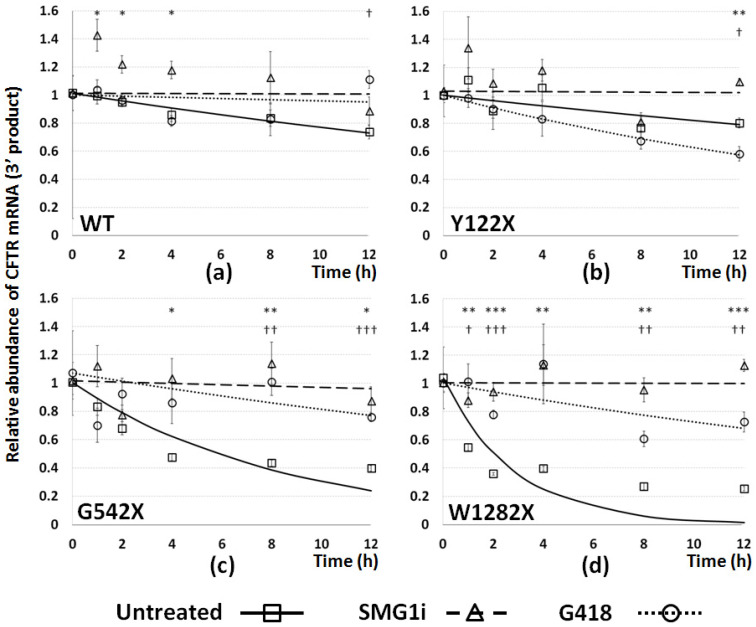
Effect of PTC mutations and of treatments on CFTR mRNA stability: 3′ end. Degradation curves for CFTR mRNA were generated by RT-qPCR using the 3′ CFTR primer pair following transcriptional shutdown using Actinomycin-D (5 µg/mL) in 16HBE cells expressing (**a**) wt-, (**b**) Y122X-, (**c**) G542X-, or (**d**) W1282X-CFTR. RNA was extracted at timepoints between 0 and 12 h. Relative abundance of CFTR mRNA is represented on the y-axis, and time (h) following Actinomycin-D administration on the x axis. At the time of initial transcriptional shutdown, cells were either untreated (squares and solid regression curves) or treated with G418 (500 µg/mL for 24 h: circles and dotted line) or SMG1i (1 mM, 24 h: triangles and dashed line). Observed data values are represented by shapes (*n* = 3 ± SEM), and exponential degradation curves generated by best fit analysis overlaid. Significant differences between untreated cells and SMG1i (asterisks) and G418 (crosses) treatments are shown (*p* < 0.05, 0.01, or 0.001).

Shutdown of mRNA transcription by treatment with Actinomycin-D in 16HBE cells was performed at various times and degradation curves were produced following PTC readthrough and NMD inhibition treatments, as above. The curves shown in Figure 5 were produced by RT-qPCR using the 3′ CFTR primer pair, and therefore represent degradation of the 3′ region of the CFTR mRNA. The data suggest that in untreated cells, the presence of specific PTC mutations can lead to marked differences in the stability of CFTR mRNA. For wt- and Y122X-CFTR, the degradation curves were almost flat over 12 h of treatment with Actinomycin-D, suggesting that these mRNAs were very stable, with half-lives of more than 12 h (Figure 5a,b). This suggests that mRNA bearing the Y122X mutation is somehow evading NMD, perhaps through reinitiation of translation, as discussed elsewhere [34]. For the remaining PTC mutations, however, the curves fell steeply, with half-lives of approximately 6 h and 2 h for G542X-CFTR and W1282X-CFTR mRNAs, respectively (Figure 5c,d). Treatment with G418 and SMG1i had no significant effect on degradation curves for the wt- and Y122X genotypes, although there is evidence of a destabilization of Y122X mRNA by G418, which might warrant further investigation. In the case of G542X and W1282X, both treatments resulted in a flattening of degradation curves, bringing the half-lives back to values close to those of wt-CFTR. For both the latter mutations, SMG1i had a more stabilizing effect than G418, resulting in a flatter curve, i.e., a longer transcript half-life.

**Figure 6:**
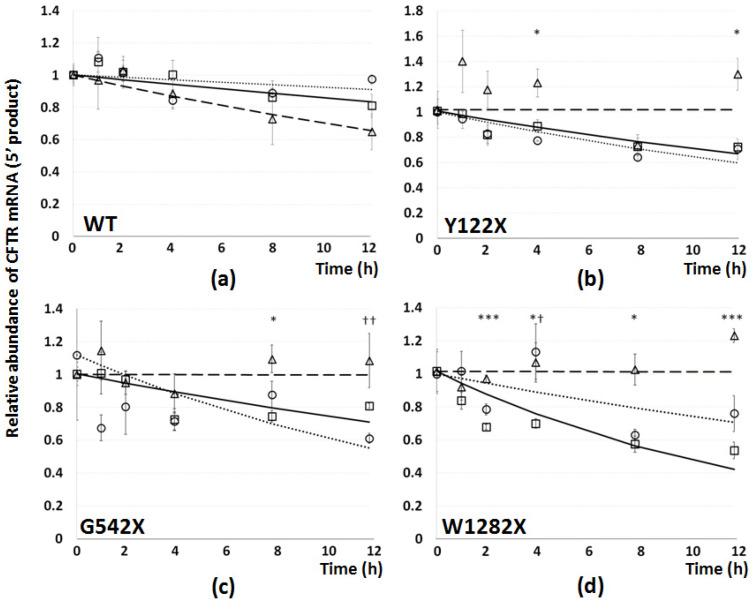
Effect of PTC mutations and of treatments on CFTR mRNA stability: 5′ end. Degradation curves for CFTR mRNA were generated by RT-qPCR using the 5′ CFTR primer pair following transcriptional shutdown using Actinomycin-D, exactly as for the 3′ primer pair (Figure 5), in 16HBE cells expressing (**a**) wt-, (**b**) Y122X-, (**c**) G542X-, or (**d**) W1282X-CFTR. Relative abundance of CFTR mRNA is represented on the y-axis, and time (h) following Actinomycin-D administration on the x axis. Cells were either untreated (squares and solid regression curves) or treated with G418 (500 µg/mL for 24 h: circles and dotted line) or SMG1i (1 mM, 24 h: triangles and dashed line). Observed data values are represented by shapes (*n* = 3 ± SEM), and exponential degradation curves generated by best fit analysis overlaid. Significant differences between untreated cells and SMG1i (asterisks) and G418 (crosses) treatments are shown (*p* < 0.05, 0.01, or 0.001).

We also produced degradation curves using the 5′ CFTR primer pair (Figure 6), for all genotypes. As observed for the 3′ degradation curves, W1282X mRNA was found to have a steeper degradation curve than G542X mRNA, but the degradation for both G542X and W1282X mutations occurred at a lower rate at the 5′ end than at the 3′ end of the CFTR mRNA, resulting in much higher estimates for half-life (greater than 12 h for G542X, approximately 10 h for W1282X). In this assay, NMD inhibition by SMG1i and especially PTC readthrough by G418 were not found to have such consistently significant stabilizing effects as at the 3′ end, and G418 even aggravated the instability by the 12 h timepoint in G542X cells.

## 4. Discussion

Mutations in class I, which includes nonsense or premature stop codon (PTC) mutations [4], some of which are among the most common mutations in CF, have been largely bypassed by the recently approved CFTR corrector therapies [19]. Ongoing efforts to rectify this situation include the search for novel small molecules that induce translational readthrough [35], or combinations of PTC readthrough with NMD inhibition [9,20] in parallel with more directed approaches including gene therapy or editing [36], or the blocking of exon junction complex (EJC) deposition at specific exon–exon boundaries using antisense oligonucleotides (ASOs) [37]. In the present study, we provide a more detailed characterization of PTC-containing CFTR mRNA—namely, its relative abundance, integrity, and stability—in an attempt to elucidate some of the challenges facing strategies including PTC readthrough and NMD inhibition.

Our data show that in 16HBE cells stably expressing three different PTCs, these nonsense mutations trigger NMD as expected, resulting in a lower-than-normal abundance for the PTC-bearing mRNAs (Figure 2a). Our data also provide some evidence for mutation-specific variations in the extent of NMD detected, in support of previous findings [10,11]. Levels of CFTR-mRNA-bearing PTCs were partially restored by PTC readthrough (G418) and to a much greater extent by NMD inhibition (SMG1i: Figure 2b–e). These effects are thought to take place via separate mechanisms of action, with G418 essentially sidestepping the NMD trigger by removing the EJC “roadblock” following translational readthrough [15], in contrast to the direct inhibition of an NMD protein on the part of SMG1i. Interestingly, the extent of restoration of CFTR mRNA abundance was mutation-dependent, with W1282X-CFTR levels undergoing a 12-fold increase upon SMG1i administration (for the 3′ CFTR product) compared with approximately 6-fold and 2-fold increases for G542X and Y122X-CFTR, respectively, under the same conditions. The increased CFTR mRNA levels stimulated by SMG1i treatment were found to be up to 4-fold higher than wt-CFTR values in untreated cells (Figure S1). Thus, for the 3′ product at least, not unexpectedly, the greater the initial effect of NMD, the greater the subsequent restoration of mRNA abundance by the same NMD inhibition treatment. This was not found to be the case following treatment with G418, which is one of the most commonly used experimental PTC readthrough compounds [22], and the distinct effects of the two compounds highlight their different modes of action: G418 is only able to stabilize intact mRNA molecules that have not already been targeted to NMD, by translational readthrough of the PTC, whereas SMG1i, by directly affecting the NMD machinery, enhances the accumulation of PTC-bearing mRNAs. This might explain any synergistic effect of combining readthrough and NMD inhibition [20].

Western blot data show that the restoration of mRNA expression by PTC readthrough and/or NMD inhibition was not, however, accompanied by equivalent restoration of significant amounts of full-length protein (Figure 3). Two antibodies were used to probe CFTR, and only in the case of W1282X—which has previously been shown to produce a truncated protein with some chloride channel function, even without PTC readthrough [5]—was there evidence that inhibition of NMD by SMG1i stabilized the mRNA, thereby allowing some more (truncated) protein to be produced. The presence of truncated Y122X and G542X proteins following treatment with SMG1i, although expected, was not detected, which we attribute to the quality of the TJA9 antibody, the only one with an epitope predicted to be present in these two proteins. Interestingly, a recent report demonstrated functional rescue of both Y122X and G542X mutants in polarized cultures of 16HBE cells by G418, with no additive effect of SMG1i, whereas for W1282X G418 it was ineffective, functional rescue in this case being stimulated by a combination of SMG1i and CFTR correctors [38]. This supports our conclusions for W1282X, but in no case did readthrough by G418 itself have an appreciable effect on protein production in our study, with the G542X CFTR being utterly unresponsive to both treatments at the protein level. A faint signal seen upon quantification of Y122X CFTR (Figure 3B) may reflect the effect of alternative initiation of translation, previously shown to be possible at methionines M150, M152, and M154 encoded in exon 4 of *CFTR* [34], which can produce CFTR protein of negligible function (and low stability), truncated at the N-terminus. 

The absence of appreciable differences in CFTR protein expression upon NMD inhibition and PTC readthrough might partly be explained by our data on CFTR mRNA integrity, namely, that there were always more abundant 3′ mRNA transcripts than 5′ transcripts, even for wt-CFTR, judging by 3′:5′ ratios (Figure 4). This 3′ bias was also found, to a lesser extent, in native respiratory cells (Supplementary Materials, Figure S2), supporting its importance as a potential factor in the efficacy of PTC readthrough approaches. Our use of random primers to generate cDNA bypasses any end bias effect that might be seen with reverse transcription using oligo-dT primers [39], where some cDNA fragments might not include the 5′ end region, either because of intermediate cleavage, inefficient reverse transcription due to secondary mRNA structure, or the great length of the CFTR transcript. With random primers, even cleaved 5′ transcripts containing the 5′ PCR product region should be represented in the cDNA. Therefore, our data suggest that the 5′ region of CFTR mRNA, including the translation initiation codon, being less abundant that the 3′ region in untreated cells, is fundamentally more prone to degradation by both NMD and, given the similar ratios for wt-CFTR, likely also by non-NMD mechanisms. Therefore, despite previous studies that have measured significant quantities of PTC bearing mRNA escaping NMD (e.g., 30% of the levels measured for non PTC or F508del transcripts [11]), the value of translatable mRNA (i.e., containing the ATG codon and significant amounts of subsequent sequence) must in effect be lower than those measurements. Furthermore, much of the detected 5′ transcript containing the start codon may be cleaved part of the way and partially degraded [16,17], therefore not providing a suitable substrate for full-length protein translation. This applies also to the higher levels of PTC mRNA following PTC readthrough and NMD inhibition, which are also characterized by elevated 3′:5′ ratios. This combination of NMD-mediated reduction of PTC CFTR mRNA with preferential degradation of the 5′ end may reduce the effective abundance of these transcripts to levels far below those measured and explain the observed lack of rescued CFTR protein. For any therapeutic strategy with functional significance in terms of protein production, any boost in mRNA expression might therefore need to promote a shift towards stabilization of the 5′ region. Although we demonstrate (Figure S2) that such ratios may vary under different conditions and in different cell types, we believe that our overall data provide an important caveat that the effective levels of PTC-bearing mRNAs may not be as high as those measured by any particular pair of primers in RT-qPCR. Further studies on integrity of PTC-bearing CFTR mRNA might be designed to use different combinations of primers, including those in the middle of the mRNA, and other methods such as RNAseq should also be considered. The development of single-mRNA-molecule imaging techniques may also clarify the dynamics of mRNA turnover, with and without NMD [40], which may soon allow for a more accurate estimate of the mRNA available for protein translation.

The degradation curves generated following transcriptional shutdown by Actinomycin-D suggest that stability of the 3′ mRNA region is much reduced for the G542X and W1282X mutations (Figure 5c,d), but not for Y122X, which had a shallow curve similar to that seen for wt-CFTR (Figure 5a,b). This suggests that this mutation does not induce the same level of NMD-mediated instability as the other two PTC mutations. Significant stabilizing effects by G418 and especially SMG1i were seen on both G542X and W1282X mRNAs, as expected, given the data on their respective abundance. Evidence for the differential processing of different PTC mutations at the mRNA level is provided by the destabilizing effect of G418 on Y122X: if Y122X escapes NMD due to translation reinitiation at M150 or M152 [34], the introduction of G418 may block the reinitiation by reading through the PTC, bypassing the cell’s own solution to this particular mutation. Interestingly, given the 3′:5′ ratios, degradation curves generated from the same samples using the 5′ primer set showed that the stability of the 5′ region of CFTR mRNA is greater in untreated cells than the 3′ region for both G542X and W1282X (Figure 6), and is also stabilized by NMD inhibition following SMG1i administration.

Stability of eukaryotic mRNA is rather variable, with half-lives ranging from a few minutes to a few days [30,41]. Furthermore, the half-life can vary widely for the same gene under different conditions and can also be significantly affected by the method used to measure it [42]. In mouse embryonic stem (ES) cells, the wt-Cftr mRNA has an intermediate half-life, ranging from 4.3–9.3 h, with the greater figure seen in more differentiated cells [43]. Our degradation curves, produced in human bronchial epithelial cell lines, suggest that wt-CFTR mRNA has greater stability, and are closer to values observed in HT29 and Calu-3 cells [44], providing evidence that a cell-specific posttranscriptional environment determines CFTR mRNA stability. In any case, the steady-state pool of CFTR mRNA available in a given cell type under a given condition will be the result of a combination of factors inducing degradation. These include the presence or absence of mRNA stability determinant sequences (mainly in the 3′ region) and their binding factors [42], posttranscriptional mRNA regulatory cascades linked to inflammation [44], which are likely to become more important in vivo, and other factors including miRNAs, which are only now starting to be investigated [45]. Thus, the presence of a PTC in the mRNA only accelerates what is already a complex process of degradation.

In conclusion, our data suggest that, based on measurements of CFTR mRNA abundance, integrity, and stability, the optimized rescue of PTC mutations has to take into account (1) the different steady-state levels of CFTR mRNA associated with each specific PTC mutation; (2) differences in abundance between the 3′ and 5′ regions of CFTR mRNA, even following PTC readthrough or NMD inhibition; and (3) variable effects on CFTR mRNA stability for each specific PTC mutation.

## Supplementary Materials

The following are available online at https://www.mdpi.com/article/10.3390/genes12111810/s1, Figure S1: Abundance of CFTR mRNA: overall comparison to wt-CFTR control baseline. Figure S2: Ratios of abundance of 3′ and 5′ CFTR mRNA in native respiratory tissues, measured by RT-qPCR.

## Abbreviations

RT-qPCR—quantitative reverse transcription polymerase chain reaction; PTC—premature termination codon; NMD—nonsense-mediated decay; WB—Western blot; wt—wild type.
