# Supplementary material for: Integrity and Stability of PTC Bearing CFTR mRNA and Relevance to Future Modulator Therapies in Cystic Fibrosis
Source: Genes (Basel). 2021 Nov 18;12(11):1810. doi: 10.3390/genes12111810 (PMC8621375; doi:10.3390/genes12111810)
Supplement: Supplementary file 1 [file genes-12-01810-s001.zip › genes-1421466-supplementary.pdf]

Supplementary Data

Figure S1

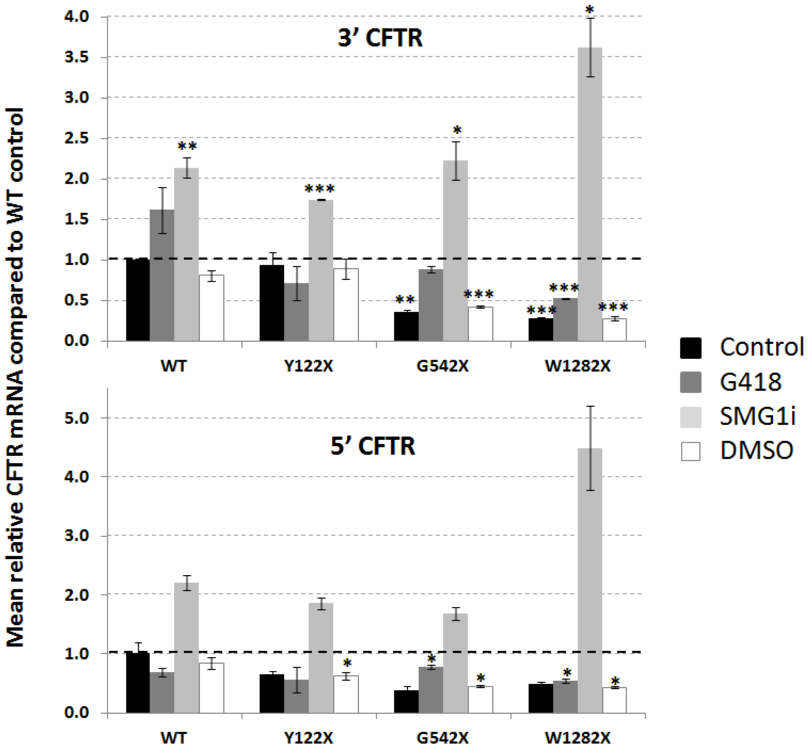

Figure S1. Abundance of CFTR mRNA: overall comparison to wt-CFTR control baseline. Graphs show relative quantification of CFTR mRNA amplified by RT-qPCR using primers designed at 3' and 5' ends (top and bottom panels, respectively), in 16HBE cells with four different CFTR genotypes (WT, Y122X, G542X, and W1282X), and under four different conditions (Untreated control cells; G418, 250 µg/ml, 24h; SMG1i, 0.5 µM, 24h; DMSO 0.5%, 24h). All conditions are compared to wt-CFTR control values, to provide an estimate of overall mRNA values to a standard baseline in untreated wild type cells. Values shown are means (n=3, except for wt-CFTR Ctrl: n=2) ±SEM, and significant differences from respective control conditions are indicated by asterisks (*p<0.05; **p<0.01; ***p<0.001).

Figure S2

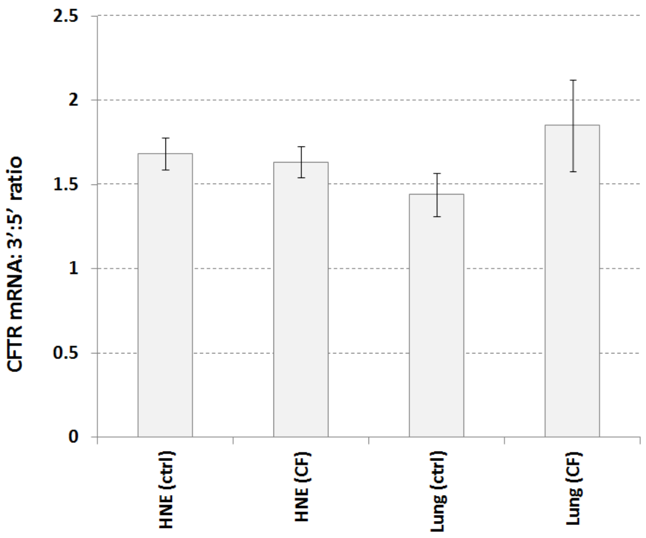

Figure S2. Ratios of abundance of 3' and 5' CFTR mRNA in native respiratory tissues, measured by RT-qPCR. Ratios of 3' to 5' transcript abundance were determined for human nasal epithelial cells (HNE) and bronchial tissue samples (Lung) obtained from healthy controls or donors (ctrl) or individuals with cystic fibrosis (CF). HNE samples were obtained by nasal brushing from n=4 healthy controls, and n=6 individuals with CF (PTC/F508del heterozygous CFTR genotype). Lung samples were obtained from n=4 healthy donors and n=4 individuals with CF (F508del homozygous CFTR genotype). RNA was extracted, cDNA reverse transcribed, and relative quantification of 3' *vs.* 5' CFTR transcripts performed using RT-qPCR, as described in main text (see Fig. 4). Values shown are means ±SEM. All samples were obtained following appropriate consent procedures and ethical approval.
